# Operational matrices based on the shifted fifth-kind Chebyshev polynomials for solving nonlinear variable order integro-differential equations

**DOI:** 10.1186/s13662-021-03588-2

**Published:** 2021-10-02

**Authors:** H. Jafari, S. Nemati, R. M. Ganji

**Affiliations:** 1 Department of Applied Mathematics, Faculty of Mathematical Sciences, University of Mazandaran, P.O. Box: 47416-95447, Babolsar, Iran; 2 Department of Mathematical Sciences, University of South Africa, UNISA0003, Pretoria, South Africa; 3 Department of Medical Research, China Medical University Hospital, China Medical University, Taichung, 110122 Taiwan

**Keywords:** Shifted fifth-kind Chebyshev polynomials, Variable order, Nonlinear integro-differential equations, Operational matrix, Convergence analysis

## Abstract

In this research, we study a general class of variable order integro-differential equations (VO-IDEs). We propose a numerical scheme based on the shifted fifth-kind Chebyshev polynomials (SFKCPs). First, in this scheme, we expand the unknown function and its derivatives in terms of the SFKCPs. To carry out the proposed scheme, we calculate the operational matrices depending on the SFKCPs to find an approximate solution of the original problem. These matrices, together with the collocation points, are used to transform the original problem to form a system of linear or nonlinear algebraic equations. We discuss the convergence of the method and then give an estimation of the error. We end by solving numerical tests, which show the high accuracy of our results.

## Introduction

Fractional calculus, which is a generalization of differentiation and integration from integer order to any arbitrary order, has attracted numerous researchers in engineering and science [1–13]. Different problems in variety fields of applied science can be described by fractional derivatives (FDs). Recently, Khan and Atangana [14] have modeled the dynamics of novel coronavirus (2019-nCov) with FD. Also, Ganji et al. [15] have simulated a mathematical model of brain tumor involving fractional derivative.

Since the order of fractional integrals and derivatives may take any arbitrary value, a new extension of these operators has been proposed such that the order of these operators is not a constant but a function of some independent variables such as time or space. In 1993, Samko and Ross [16] were the first researchers who have suggested the study of VO operators. Then theory-based studies of VO calculus have been more deeply investigated by Lorenzo and Hartley [17]. Soon after, many definitions of VO derivative operators have been introduced by some researchers such as Riemann–Liouville (RL) [18, 19], Lorenzo–Hartley [17], Coimbra [20], and Caputo [2, 21] derivatives. These operators have been used to describe some models in a variety of science fields including biochemical tumorous bone remodeling models [22], characterizing the dynamics of van der Pol oscillators [23]; see also [24, 25]. Since in this type of problems, we confront with a kernel of VO [26], computing analytical solutions is very difficult. Hence developing effective numerical techniques for finding approximate solution for such problems is very important and necessary. In recent years, many researchers have proposed different schemes to solve this kind of problems. To mention a few, we refer to [27–30], where the authors have applied operational matrices based on various polynomials to get approximate solutions of different problems of VO.

A significant aim of this research is to express a numerical scheme to solve the following VO-IDEs:

1 $$ \begin{gathered} {{}^{C}D^{\upsilon (t)}_{t} }z(t)= \lambda F \biggl(t,z(t), \int _{0}^{1}K_{1}(t, \tau ) \phi _{1} \bigl(\tau ,z(\tau ) \bigr) \,d\tau , \int _{0}^{t}K_{2}(t, \tau ) \phi _{2} \bigl(\tau ,z(\tau ) \bigr) \,d\tau \biggr),\\ \quad t\in [0,1], \end{gathered} $$

with initial conditions

2 $$\begin{aligned} z^{(i)}(0)=\mathfrak{a}_{i},\quad i =0,1,\ldots ,p-1, \end{aligned}$$

where $p-1<\upsilon (t)\leq p $, *p* is a positive integer number, *F* is a given continuous function, *λ* and $\mathfrak{a}_{i}$, $i=0,1,\ldots ,p-1 $, are real constants, $K_{1}$, $K_{2}$, $\phi _{1}$, and $\phi _{2}$ are given known functions, $z(t) $ is the unknown solution, and ${{}^{C}D^{\upsilon (t)}_{t} }$ denotes the variable-order derivative operator in the Caputo sense.

Many researchers in various fields of science employ orthogonal basis functions to get approximate solutions for many problems [31–33]. The fifth-kind Chebyshev polynomials consist a special class of symmetric orthogonal polynomials, which are created with the help of the extended Sturm–Liouville theorem for symmetric functions. In this work, with the help of these polynomials, we reduce problem (1)–(2) to the solution of a system of nonlinear algebraic equations, which greatly simplifies the problem under study.

The design of this research is as follows. In Sect. 2, we introduce some essential definitions of variable fractional calculus and some basic properties of the SFKCPs. Section 3 is devoted to proposing a numerical scheme to solve problem (1)–(2). In Sect. 4, we study an error bound of the proposed scheme. Section 5 includes some examples. In the end, we give concluding remarks in Sect. 6.

## Perliminaries

In this section, we present the definitions of VO RL-integral and Caputo derivative. Then, some basic properties of the SFKCPs are given which are used later.

### VO fractional calculus

#### Definition 2.1

(See [34])

Let $p-1<\upsilon (t)\leq p$ and $z\in C[0,1] $. The RL-integral and Caputo derivative of VO $\upsilon (t)$ are, respectively, defined by

$$\begin{aligned}& {{}^{R L} I}_{t}^{\upsilon (t)} z(t)= \frac{1}{\Gamma (\upsilon (t))} \int _{0}^{t} (t-\tau )^{\upsilon (t)-1} z(\tau ) \,d \tau , \\& {{}^{C}D^{\upsilon (t)}_{t} } z(t)= \frac{1}{\Gamma (p-\upsilon (t))} \int _{0}^{t} (t-\tau )^{p-\upsilon (t)-1} z^{(p)}(\tau ) \,d\tau . \end{aligned}$$

Two main properties of these operators are given as follows:

3 $$\begin{aligned}& {{}^{C}D^{\upsilon (t)}_{t}} t^{\zeta }= \textstyle\begin{cases} \frac{\Gamma (\zeta +1)}{\Gamma (\zeta -\upsilon (t)+1)}t^{\zeta - \upsilon (t)},& \zeta \in \mathbb{N} \text{ and } \zeta \geq \lceil \upsilon (t)\rceil \text{ or } \zeta \notin \mathbb{N} \text{ and } \zeta > \lfloor \upsilon (t)\rfloor , \\ 0,& \zeta \in \mathbb{N}\cup \lbrace 0\rbrace \text{ and } \zeta < \lceil \upsilon (t)\rceil , \end{cases}\displaystyle \\& {{}^{C}D^{\upsilon (t)}_{t}}z(t)={{}^{R L} I^{p-\upsilon (t)}_{t}} \bigl(z^{(p)}(t) \bigr). \end{aligned}$$

### Definition of the SFKCPs and function approximation

The SFKCPs on the interval $[0,1] $ are defined by [28, 35]

$$\begin{aligned} \mathcal{C}_{m}^{*}(t)=\mathcal{C}_{m}(2t-1), \quad m=0,1,2,\ldots , \end{aligned}$$

where $\mathcal{C}_{m}(t) $ is the fifth-kind Chebyshev polynomial defined on $[-1,1] $ as follows:

$$\begin{aligned} \mathcal{C}_{m}(t)=\frac{1}{\sqrt{\delta _{m}}}\overline{ \mathcal{B}}^{(-3,2,-1,1)}_{m}(t), \end{aligned}$$

where

$$ \delta _{m}= \textstyle\begin{cases} \frac{\pi }{2^{2m+1}},& m \text{ is even}, \\ \frac{\pi (m+2)}{m 2^{2m+1}},& m \text{ is odd}, \end{cases} $$

and

$$\begin{aligned}& \overline{\mathcal{B}}_{m}^{(v,w,r,s)}(t)= \Biggl( \prod_{k=0}^{\lfloor \frac{m}{2}\rfloor -1} \frac{(2k+(-1)^{m+1}+2)s+w}{(2k+(-1)^{m+1}+2\lfloor \frac{m}{2}\rfloor )r+v} \Biggr) \mathcal{B}_{m}^{(v,w,r,s)}(t), \end{aligned}$$

with

$$\begin{aligned} \mathcal{B}_{m}^{(v,w,r,s)}(t)= \sum_{j=0}^{\lfloor \frac{m}{2}\rfloor } \Biggl(\binom{\lfloor \frac{m}{2}\rfloor }{j} \Biggl( \prod_{k=0}^{\lfloor \frac{m}{2}\rfloor -j-1} \frac{(2k+(-1)^{m+1}+2\lfloor \frac{m}{2}\rfloor )r+v}{(2k+(-1)^{m+1}+2)s+w} \Biggr) t^{m-2j} \Biggr). \end{aligned}$$

Furthermore, the analytic form of the SFKCPs of degree *m* is given by

$$\begin{aligned}& \mathcal{C}_{m}^{*}(t)=\sum _{l=0}^{m}\varsigma _{l,m} t^{l}, \end{aligned}$$

where

4 $$\begin{aligned} \varsigma _{l,m}=\frac{2^{2l+\frac{3}{2}}}{\sqrt{\pi }(2l)!} \textstyle\begin{cases} 2 \sum_{k=\lfloor \frac{l+1}{2}\rfloor }^{\frac{m}{2}} \frac{(-1)^{\frac{m}{2}+k-l} k \varepsilon _{k} (2k+l-1)!}{(2k-l)!},& m \text{ is even}, \\ \frac{1}{\sqrt{m(m+2)}} \sum_{k=\lfloor \frac{l}{2} \rfloor }^{\frac{m-1}{2}} \frac{(-1)^{\frac{m+1}{2}+k-l}(2k+1)^{2} (2k+l)!}{(2k-l+1)!},& m \text{ is odd}, \end{cases}\displaystyle \end{aligned}$$

and

$$\begin{aligned} \varepsilon _{k}= \textstyle\begin{cases} \frac{1}{2},& k=0, \\ 1,& k>0. \end{cases}\displaystyle \end{aligned}$$

Also, the orthogonality condition is given for these polynomials as follows:

$$\begin{aligned} \int _{0}^{1} w^{*}(t) \mathcal{C}_{r}^{*}(t) \mathcal{C}_{s}^{*}(t) \,dt= \textstyle\begin{cases} 1,&r= s, \\ 0,&r\neq s, \end{cases}\displaystyle \end{aligned}$$

where $w^{*}(t)= \frac{(2t-1)^{2}}{\sqrt{t-t^{2}}} $.

#### Lemma 2.1

(See [35])

*The SFKCPs satisfy the following boundedness property on*$[0,1] $*for all*$s\geq 0 $:

$$\begin{aligned} \bigl\vert \mathcal{C}_{s}^{*}(t) \bigr\vert < \sqrt{ \frac{2}{\pi }}(s+2),\quad \forall t\in [0,1]. \end{aligned}$$

Suppose that $r_{1}, r_{2}\in L^{2}_{w^{*}}(0,1)$. Then the inner product and norm in $L^{2}_{w^{*}}(0,1)$ are, respectively, defined by

$$\begin{aligned}& \langle r_{1},r_{2}\rangle _{w^{*}}= \int _{0}^{1} w^{*}(t)r_{1}(t)r_{2}(t) \,dt, \\& \Vert r_{1} \Vert _{2}=\sqrt{\langle r_{1},r_{1}\rangle _{w^{*}}}. \end{aligned}$$

Any arbitrary function $z(t)\in L_{w^{*}}^{2}(0,1)$ can be expanded by the SFKCPs as

5 $$\begin{aligned} z(t)=\sum_{i=0}^{\infty }z_{i} \mathcal{C}_{i}^{*}(t). \end{aligned}$$

By considering only the first $M+1$ terms in (5), we can approximate $z(t)$ as

$$\begin{aligned} z(t)\simeq z_{M}(t)=\sum _{i=0}^{M}z_{i}\mathcal{C}_{i}^{*}(t)=Z^{T} \varphi (t), \end{aligned}$$

where

$$\begin{aligned} \varphi (t)=\bigl[\mathcal{C}_{0}^{*}(t), \mathcal{C}_{1}^{*}(t),\ldots , \mathcal{C}_{M}^{*}(t) \bigr]^{T}, \end{aligned}$$

and in the vector $Z=[z_{0},z_{1},\ldots ,z_{M}]^{T}$, the entries $z_{i}$, $i=0,1,\ldots ,M $, are given by

6 $$\begin{aligned} z_{i}= \int _{0}^{1}w^{*}(t) z(t) \mathcal{C}_{i}^{*}(t)\,dt. \end{aligned}$$

In a similar way, a bivariate function $f(t,\tau )\in L^{2}_{w^{*}} ((0,1)\times (0,1) ) $ can be approximated based on the SFKCPs as

$$\begin{aligned} f(t,\tau )\simeq \sum_{i=0}^{M}\sum _{j=0}^{M}f_{ij} \mathcal{C}^{*}_{i}(t) \mathcal{C}^{*}_{j}( \tau )=\varphi ^{T}(t) F \varphi (\tau ), \end{aligned}$$

where *F* is an $(M+1)\times (M+1)$ matrix given by

$$\begin{aligned} F= \bigl\langle \varphi (t),\bigl\langle f(t,\tau ),\varphi (\tau ) \bigr\rangle _{w^{*}} \bigr\rangle _{w^{*}}. \end{aligned}$$

We can consider the vector $\varphi (t)$ in a matrix form as

7 $$\begin{aligned} \varphi (t)=AT_{M}(t), \end{aligned}$$

where $A=[a_{i,j}]$, $i,j=0,1,\ldots ,M $, with

$$\begin{aligned} a_{i,j}= \textstyle\begin{cases} \varsigma _{i,j}, &i\geq j, \\ 0,&i< j, \end{cases}\displaystyle \end{aligned}$$

$\varsigma _{i,j} $ are given by (4), and

$$\begin{aligned} T_{M}(t)=\bigl[1,t,\ldots ,t^{M}\bigr]^{T}. \end{aligned}$$

#### Theorem 2.1

(See [35])

*Suppose that*$z(t)\in L^{2}_{w^{*}}(0,1) $*with*$\vert z^{(3)}(t)\vert \leq \theta $. *Let*$\sum_{i=0}^{\infty }z_{i}\mathcal{C}_{i}^{*}(t)$*be its expansion using the SFKCPs*. *Then*, *for*$i>3$, *the coefficient*$z_{i}$*is bounded as*

$$\begin{aligned} \vert z_{i} \vert < \frac{\sqrt{2\pi } \theta }{2 i^{3}}. \end{aligned}$$

#### Lemma 2.2

*Consider the basis vector*$\varphi (t)$*defined by* (7). *By applying the first*-*order derivative on this vector we get*

$$\begin{aligned} \frac{d}{dt}\varphi (t)= D\varphi (t), \end{aligned}$$

*where* *D* *is the operational matrix of derivative based on the SFKCPs given by*

$$\begin{aligned} D=A \begin{bmatrix} 0&0&0&\cdots &0&0 \\ 1&0&0&\cdots &0&0 \\ 0&2&0&\cdots &0&0 \\ \vdots &\vdots &\vdots &\vdots &\vdots &\vdots \\ 0&0&0&\cdots &M&0 \end{bmatrix} A^{-1}. \end{aligned}$$

*Also*, *for* $m\geq 2 $, *we can write*

8 $$\begin{aligned} \frac{d^{m}}{dt^{m}}\varphi (t)=D^{m} \varphi (t). \end{aligned}$$

#### Proof

It can be easily proved in a similar way as that of the corresponding theorem in [36]. □

#### Lemma 2.3

*For the vector*$\varphi (t) $*given by* (7), *the dual operational matrix* *Q* *is given by*

9 $$\begin{aligned} \int _{0}^{1}\varphi (\tau )\varphi ^{T}( \tau )\,d\tau =A \biggl( \int _{0}^{1}T_{M}( \tau )T_{M}^{T}(\tau )\,d\tau \biggr)A^{T}=Q, \end{aligned}$$

*where* $Q=AHA^{T}$ *with the well*-*known Hilbert matrix* *H*.

#### Proof

The proof process is similar to that given in [36]. □

#### Lemma 2.4

*The integral of the vector*$\varphi (t) $*given by* (7) *can be approximated as*

10 $$\begin{aligned} \int _{0}^{t}\varphi (\tau )\,d\tau \simeq P\varphi (t), \end{aligned}$$

*where* *P* *is called the operational matrix of integration for the SFKCPs*.

#### Proof

Using (7), we write

$$\begin{aligned} \int _{0}^{t}\varphi (\tau )\,d\tau =A \int _{0}^{t}T_{M}(\tau )\,d\tau =A B T^{*}(t), \end{aligned}$$

where $B=[b_{i,j}]$, $i,j=0,1,\ldots ,M $, is an $(M+1)\times (M+1) $ matrix with elements

$$\begin{aligned} b_{i,j}= \textstyle\begin{cases} \frac{1}{i+1},&i=j, \\ 0,&i\neq j, \end{cases}\displaystyle \end{aligned}$$

and

$$\begin{aligned} T^{*}(t)= \begin{bmatrix} t,t^{2},\ldots ,t^{M+1} \end{bmatrix} ^{T}. \end{aligned}$$

Now, by approximating $t^{k}$, $k=1,2,\ldots ,M+1 $, in terms of the SFKCPs using (7), we have

$$\begin{aligned} \textstyle\begin{cases} t^{k}=A^{-1}_{k+1}\varphi (t),&k=1,2,\ldots ,M, \\ t^{M+1}=\mathfrak{L}^{T}\varphi (t), \end{cases}\displaystyle \end{aligned}$$

where $A^{-1}_{i}$, $i=2,3,\ldots ,M+1$, is the *i*th row of the matrix $A^{-1}$, and $\mathfrak{L}=\langle t^{M+1}, \varphi (t)\rangle _{w^{*}} $. Then, we get

$$\begin{aligned} T^{*}(t)=E\varphi (t), \end{aligned}$$

where $E= [ A_{2}^{-1},A_{3}^{-1},\ldots ,A_{M+1}^{-1},\mathfrak{L}^{T} ] ^{T} $. Therefore by taking $P=ABE $, we complete the proof. □

#### Lemma 2.5

*Suppose*$Z= [ z_{0},z_{1},\ldots , z_{M} ] ^{T}$. *ThenẐis the operational matrix of product whenever*

11 $$\begin{aligned} \varphi (t)\varphi ^{T}(t)Z\simeq \widehat{Z}\varphi (t). \end{aligned}$$

#### Proof

According to (7) and expanding the function $\mathcal{C}_{i}^{*}(t)\mathcal{C}_{j}^{*}(t)$, $i,j=0,1,\ldots ,M $, we have

$$\begin{aligned} \mathcal{C}_{i}^{*}(t)\mathcal{C}_{j}^{*}(t) \simeq \sum_{m=0}^{i+j}c_{m} \mathcal{C}_{m}^{*}(t), \end{aligned}$$

where $c_{m} $, $m=0,1,\ldots ,i+j$, can be computed as

$$\begin{aligned} c_{m}= \sum_{k=0}^{i} \sum _{l=0}^{j} \sum _{s=0}^{m}\varsigma _{i,k} \varsigma _{j,l} \varsigma _{m,s} \int _{0}^{1}w^{*}(t)t^{k+l+s} \,dt=\Delta _{i,j,m}, \end{aligned}$$

with

$$\begin{aligned} \Delta _{i,j,m}= \sum_{k=0}^{i} \sum_{l=0}^{j} \sum _{s=0}^{m} \frac{\sqrt{\pi } (3+k^{2}+s(3+s)+k(3+2s) )\Gamma (\frac{3}{2}+k+s)}{\Gamma (4+k+s)} \varsigma _{i,k} \varsigma _{j,l} \varsigma _{m,s}. \end{aligned}$$

By considering $Z= [ z_{0},z_{1},\ldots ,z_{M}] $ and (6), we have

$$\begin{aligned} \varphi (t)\varphi ^{T}(t) Z\simeq \widehat{Z}\varphi (t), \end{aligned}$$

where the elements of $\widehat{Z}=[\widehat{z_{i,j}}]$, $i,j=0,1,\ldots ,M $, are given by

$$\begin{aligned} \widehat{z_{i,j}}=\sum_{m=0}^{M} \Delta _{i,j,m} z_{m}. \end{aligned}$$

 □

#### Theorem 2.2

*Let*$\varphi (t) $*be the SFKCPs vector given in* (7), *and let* $p-1<\upsilon (t)\leq p $. *Then*

12 $$\begin{aligned} {{}^{C}D^{\upsilon (t)}_{t} }\varphi (t)=\Upsilon ^{\upsilon (t)} \varphi (t), \end{aligned}$$

*where* $\Upsilon ^{\upsilon (t)}= A\Psi ^{\upsilon (t)} A^{-1}$ *with*

13 $$\begin{aligned} \Psi ^{\upsilon (t)}=\bigl[\rho _{t}^{i,j} \bigr],\quad i,j=0,1,\ldots ,M, \end{aligned}$$

*and*

$$\begin{aligned} \rho _{t}^{i,j}= \textstyle\begin{cases} \frac{\Gamma (i+1)}{\Gamma (i+1-\upsilon (t))}t^{-\upsilon (t)},&i=j \& i\geq p, \\ 0,&\textit{otherwise}. \end{cases}\displaystyle \end{aligned}$$

#### Proof

By employing ${{}^{C}D^{\upsilon (t)}_{t} } $ to both sides of (7), we get

14 $$\begin{aligned} {{}^{C}D^{\upsilon (t)}_{t} }\varphi (t)={{}^{C}D^{\upsilon (t)}_{t} }\bigl(AT_{M}(t) \bigr)=A\bigl({{}^{C}D^{\upsilon (t)}_{t} }T_{M}(t) \bigr). \end{aligned}$$

Taking into account that $p=\lceil \upsilon (t)\rceil $ and using (3), (14) becomes

$$\begin{aligned} \begin{aligned} {{}^{C}D^{\upsilon (t)}_{t} }\varphi (t)&=A \biggl[ 0,0,\ldots ,0,\frac{\Gamma (p+1)}{\Gamma (p+1-\upsilon (t))}t^{p- \upsilon (t)},\ldots ,\frac{\Gamma (M+1)}{\Gamma (M+1-\upsilon (t))}t^{M- \upsilon (t)} \biggr] ^{T} \\ &= A\Psi ^{\upsilon (t)} T_{M}(t), \end{aligned} \end{aligned}$$

where $\Psi ^{\upsilon (t)}$ is given as (13). Therefore from (7), we get

$$\begin{aligned} {{}^{C}D^{\upsilon (t)}_{t} }\varphi (t)=\Upsilon ^{\upsilon (t)} \upsilon (t), \end{aligned}$$

with

$$\begin{aligned} \Upsilon ^{\upsilon (t)}= A\Psi ^{\upsilon (t)} A^{-1}. \end{aligned}$$

 □

## Numerical scheme

The aim of this section is to propose a numerical scheme for solving problem (1)–(2). To do this, we first consider an approximate solution of equation (1) in terms of the SFKCPs as

15 $$\begin{aligned} z(t)\simeq Z^{T}\varphi (t). \end{aligned}$$

By employing ${{}^{C}D^{\upsilon (t)}_{t} } $ to both sides of (15) and using (12), we have

16 $$\begin{aligned} {{}^{C}D^{\upsilon (t)}_{t} }z(t)= Z^{T}\Upsilon ^{\upsilon (t)} \varphi (t). \end{aligned}$$

Now we must approximate the Fredholm and Volterra parts of equation (1). To do this, the functions $K_{1}$, $K_{2} $, $\phi _{1}$, and $\phi _{2} $ are expanded using the SFKCPs as

17 $$\begin{aligned} \begin{gathered} K_{1}(t,\tau ) \simeq \varphi ^{T}(t) \mathcal{K}_{1} \varphi (\tau ), \\ K_{2}(t,\tau )\simeq \varphi ^{T}(t) \mathcal{K}_{2} \varphi (\tau ), \\ \phi _{1}\bigl(t,y(t)\bigr)\simeq H^{T} \varphi (t), \\ \phi _{2}\bigl(t,y(t)\bigr)\simeq S^{T} \varphi (t). \end{gathered} \end{aligned}$$

From (9)–(11) and (17), we obtain

18 $$\begin{aligned}& \begin{aligned}[b] \int _{0}^{1}K_{1}(t,\tau ) \phi _{1} \bigl(\tau ,y(\tau ) \bigr) \,d \tau &\simeq \int _{0}^{1}\varphi ^{T}(t) \mathcal{K}_{1} \varphi (\tau ) \varphi ^{T}(\tau ) H \,d\tau \\ &=\varphi ^{T}(t)\mathcal{K}_{1} \biggl( \int _{0}^{1}\varphi (\tau ) \varphi ^{T}(\tau ) \,d\tau \biggr) H \\ &=\varphi ^{T}(t) \mathcal{K}_{1} Q H, \end{aligned} \end{aligned}$$

19 $$\begin{aligned}& \begin{aligned}[b] \int _{0}^{t}K_{2}(t,\tau ) \phi _{2} \bigl(\tau ,y(\tau ) \bigr) \,d \tau &\simeq \int _{0}^{t}\varphi ^{T}(t) \mathcal{K}_{2} \varphi (\tau ) \varphi ^{T}(\tau ) S \,d \tau \\ &=\varphi ^{T}(t) \mathcal{K}_{2} \int _{0}^{t}\varphi (\tau ) \varphi ^{T}(\tau ) S \,d\tau \\ &=\varphi ^{T}(t) \mathcal{K}_{2} \int _{0}^{t}\widehat{S}\varphi ( \tau ) \,d\tau \\ &=\varphi ^{T}(t) \mathcal{K}_{2} \widehat{S} \int _{0}^{t}\varphi ( \tau ) \,d\tau \\ &=\varphi ^{T}(t) \mathcal{K}_{2} \widehat{S} P \varphi (t). \end{aligned} \end{aligned}$$

Substituting (15), (16), (18), and (19) into equation (1) yields

20 $$\begin{aligned} Z^{T}\Upsilon ^{\upsilon (t)}\varphi (t)- \lambda F \bigl(t,Z^{T} \varphi (t), \varphi ^{T}(t) \mathcal{K}_{1} Q H, \varphi ^{T}(t) \mathcal{K}_{2} \widehat{S} P \varphi (t) \bigr)=0. \end{aligned}$$

Taking (8) and (15) into account, we can rewrite the initial conditions (2) as follows:

21 $$\begin{aligned} Z^{T}{D^{i} \varphi (0)}- \mathfrak{a}_{i}=0,\quad i =0,1,\ldots ,p-1. \end{aligned}$$

On the other hand, by introducing the approximation $z(t)\simeq Z^{T}\varphi (t)$ into the functions $\phi _{1}$ and $\phi _{1}$ given by (17), we get

22 $$\begin{aligned} \begin{gathered} \phi _{1} \bigl(t,Z^{T}\varphi (t)\bigr)- H^{T} \varphi (t)=0, \\ \phi _{2}\bigl(t,Z^{T}\varphi (t)\bigr)- S^{T} \varphi (t)=0. \end{gathered} \end{aligned}$$

To calculate the approximate solution, we put the collocation points $\frac{r}{M+2} $ for $r=1,\ldots , M+1-p $ into equation (20). By solving simultaneously the resulting system and system (21), we get an approximation of the solution using (15).

## Convergence analysis

Here we consider the convergence of the approximate solution obtained by the proposed scheme in Sect. 3 to the analytical solution of problem (1)–(2).

### Theorem 4.1

(See [35])

*Let*$z(t)\in L^{2}_{w^{*}}(0,1) $*and suppose*$\vert z^{(3)}(t) \vert \leq \theta $*with positive constant θ*. *Suppose that the expansion ofzin terms of the SFKCPs is given by* (5). *If* $E_{n}(t)=z(t)-z_{M}(t)=\sum_{i=M+1}^{\infty }z_{i} \mathcal{C}_{i}^{*}(t) $ *is the universal error*, *then* $E_{n}(t) $ *can be evaluated as*

$$\begin{aligned} \bigl\vert E_{M}(t) \bigr\vert < \frac{3\theta }{M}. \end{aligned}$$

### Theorem 4.2

*Let*$z_{M}(t) $*be the approximate solution of problem* (1)*–*(2) *obtained by the proposed scheme in Sect*. 3, *let* $z(t)$ *be its analytical solution*, *and* $R_{M}(t) $ *be the residual error for the approximate solution*. *Also*, *suppose the Lipschitz conditions for the functions* *F*, $\phi _{1}$, *and* $\phi _{2} $ *with respect to the confirmed constants* *L*, $L_{1}$, *and* $L_{2} $, *respectively*. *Then*, *if* $z(t)$ *satisfies the conditions of Theorem *4.1, *then* $R_{M}(t) $ *tends to zero as* $M\rightarrow \infty $.

### Proof

By applying ${{}^{R L} I}_{t}^{\upsilon (t)} $ to both sides of equation (1), we can rewrite equation (1) as follows:

$$\begin{aligned} z(t)=\sum_{r=0}^{p-1}\frac{t^{r}}{r!}z^{(r)}(0)+ \lambda {{}^{R L} I}_{t}^{ \upsilon (t)} \mathcal{F}\bigl[z(t) \bigr], \end{aligned}$$

where

$$\begin{aligned}& \mathcal{F}\bigl[z(t)\bigr]=F \bigl(t,z(t),I_{1}z(t),I_{2}z(t) \bigr), \end{aligned}$$

with

$$\begin{aligned}& I_{1}z(t)= \int _{0}^{1}K_{1}(t,\tau ) \phi _{1} \bigl(\tau ,z( \tau ) \bigr) \,d\tau , \\& I_{2}z(t)= \int _{0}^{t}K_{2}(t,\tau ) \phi _{2} \bigl(\tau ,z( \tau ) \bigr) \,d\tau . \end{aligned}$$

So $z_{M}(t) $ satisfies the following equation:

$$\begin{aligned} z_{M}(t)=\sum_{r=0}^{p-1} \frac{t^{r}}{r!}z^{(r)}(0)+\lambda {{}^{R L} I}_{t}^{\upsilon (t)} \mathcal{F}\bigl[z_{M}(t) \bigr]+R_{M}(t), \end{aligned}$$

where $R_{M}(t) $ is the residual function given by

$$\begin{aligned} R_{M}(t)=z_{M}(t)-z(t)+\lambda {{}^{R L} I}_{t}^{\upsilon (t)} \bigl(\mathcal{F}\bigl[z(t)\bigr]-\mathcal{F} \bigl[z_{M}(t)\bigr] \bigr). \end{aligned}$$

Then we have

23 $$\begin{aligned} \bigl\vert R_{M}(t) \bigr\vert \leq \bigl\vert z_{M}(t)-z(t) \bigr\vert + \vert \lambda \vert \bigl\vert \mathcal{F}\bigl[z(t)\bigr]-\mathcal{F}\bigl[z_{M}(t)\bigr] \bigr\vert \biggl\vert \frac{t^{\upsilon (t)}}{\Gamma (\upsilon (t)+1)} \biggr\vert . \end{aligned}$$

Using Theorem 4.1, we have

24 $$\begin{aligned} \bigl\vert z_{M}(t)-z(t) \bigr\vert < \frac{3\theta }{M}. \end{aligned}$$

On the other hand, since $p-1<\upsilon (t)\leq p $, we have

25 $$\begin{aligned} \biggl\vert \frac{t^{\upsilon (t)}}{\Gamma (\upsilon (t)+1)} \biggr\vert \leq \frac{5}{4}. \end{aligned}$$

Since *F*, $\phi _{1}$, and $\phi _{2} $ satisfy the Lipschitz conditions, we can write

26 $$\begin{aligned} \bigl\vert \mathcal{F}\bigl[z(t)\bigr]-\mathcal{F}\bigl[z_{M}(t) \bigr] \bigr\vert \leq & L \bigl( \bigl\vert z(t)-z_{M}(t) \bigr\vert +I_{1} \bigl\vert z(t)-z_{M}(t) \bigr\vert +I_{2} \bigl\vert z(t)-z_{M}(t) \bigr\vert \bigr) \\ < & L \biggl(\frac{3\theta }{M}+\frac{3\theta }{M}k_{1}L_{1}+ \frac{3\theta }{M}k_{2}L_{2} \biggr) \\ < &\frac{3\theta L}{M} (1+k_{1}L_{1}+k_{2}L_{2} ), \end{aligned}$$

where $k_{1}=\max_{(t,\tau )\in (0,1)^{2}} \vert K_{1}(t, \tau ) \vert $ and $k_{2}=\max_{(t,\tau )\in (0,1)^{2}} \vert K_{2}(t, \tau ) \vert $. Substituting (24)–(26) into (1) yields

$$\begin{aligned} \bigl\vert R_{M}(t) \bigr\vert < \frac{3\theta }{M} \biggl(1+ \frac{5}{4} \vert \lambda \vert L (1+k_{1}L_{1}+K_{2}L_{2} ) \biggr). \end{aligned}$$

Therefore it is clear that $R_{M}(t) $ tends to zero as $M\rightarrow \infty $. □

## Numerical examples

Now we apply the proposed scheme to some examples. For solving these examples, we used the Mathematica software.

### Example 5.1

**Figure 1:**
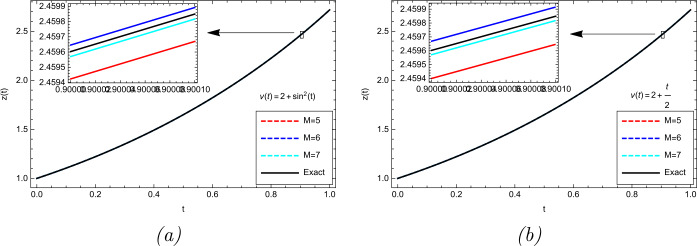
Numerical results obtained for Example 5.1. (**a**) $\upsilon (t)=\sin ^{2}(t)+2 $ (**b**) $\upsilon (t)=\frac{t}{2}+2 $

Consider the following VO problem:

$$\begin{aligned} \begin{aligned} {{}^{C}D^{\upsilon (t)}_{t} }z(t)&= \int _{0}^{1}(\tau -t) z^{2}( \tau ) \,d \tau + \int _{0}^{t}(\tau +t) z^{3}(\tau ) \,d \tau + \frac{e^{t} (\Gamma (3-\upsilon (t))-\Gamma (3-\upsilon (t),t) )}{\Gamma (3-\upsilon (t))} \\ &\quad {}+\frac{1}{36} \bigl(-13+e^{3t}(4-24t)-6t+9e^{2}(-1+2t) \bigr), \quad t\in [0,1], \end{aligned} \end{aligned}$$

under the initial conditions

$$\begin{aligned} z(0)=1,\qquad z'(0)=1,\qquad z''(0)=1, \end{aligned}$$

in which $\Gamma (\cdot ,\cdot )$ is the incomplete gamma function. We have solved this problem by different values of *M* for $\upsilon (t)=\sin ^{2}(t)+2$, $\upsilon (t)=\frac{t}{2}+2$, and the analytical solution $z(t)= e^{t} $. Figure 1 and Table 1 display the numerical results. As it can be seen from these results, the approximate solution obtained by the proposed scheme converges to the analytical one by increasing the number of basis functions.

**Table 1:** Comparison of the absolute errors (AEs) for Example 5.1

*υ*(*t*)	*t*	*M* = 5	*M* = 6	*M* = 7
2 + sin^2^(*t*)	0.1	7.19412e − 6	1.54664e − 6	2.99341e − 7
	0.3	1.10636e − 4	1.79715e − 5	2.93344e − 6
	0.5	2.31202e − 4	3.27628e − 5	7.16227e − 6
	0.7	1.83256e − 4	4.51147e − 5	1.57474e − 5
	0.9	1.79488e − 4	4.28828e − 5	3.26010e − 5
$2+\frac{t}{2}$	0.1	6.93416e − 6	1.50588e − 6	2.89671e − 7
	0.3	1.09643e − 4	1.84283e − 5	2.93573e − 6
	0.5	2.41980e − 4	3.64777e − 5	7.18866e − 6
	0.7	2.20654e − 4	5.38491e − 5	1.56380e − 5
	0.9	2.02560e − 4	6.62049e − 5	3.11399e − 5

### Example 5.2

**Figure 2:**
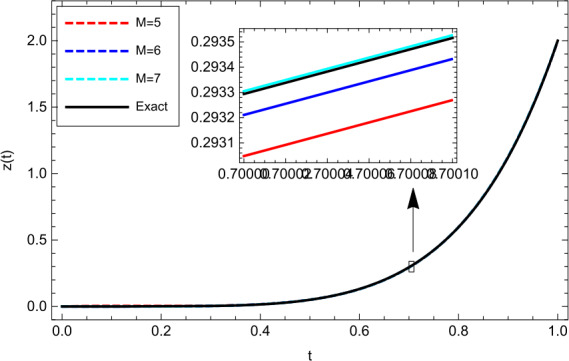
Numerical results obtained for Example 5.2

Consider the following VO problem [37]:

$$\begin{aligned} \begin{aligned} {{}^{C}D^{\upsilon (t)}_{t} }z(t)&= \int _{0}^{1}\tau \sin (t) z( \tau ) \,d\tau + \int _{0}^{t}(t-\tau ) z(\tau ) \,d\tau - \frac{16t^{\frac{27}{4}}}{621}-\frac{25 t^{\frac{41}{5}}}{1476}- \frac{299 \sin (t)}{1107} \\ &\quad {}+ \frac{\Gamma (\frac{23}{4}) t^{\frac{19}{4}-\upsilon (t)}}{\Gamma (\frac{23}{4}-\upsilon (t))}+ \frac{\Gamma (\frac{36}{5}) t^{\frac{31}{5}-\upsilon (t)}}{\Gamma (\frac{36}{5}-\upsilon (t))}, \end{aligned} \end{aligned}$$

with

$$\begin{aligned} z(0)=0, \end{aligned}$$

where $t\in [0,1] $. By considering $\upsilon (t)=t $ and carrying out the proposed scheme, the outputs obtained for this problem are depicted together with the analytical solution ($z(t)=t^{\frac{19}{4}}+t^{ \frac{31}{5}} $) in Fig. 2. From Fig. 2 it is clear that increasing the number of basis functions improves the accuracy. Furthermore, in Table 2, we have compared the outputs obtained by the proposed scheme with the method of [37] based on the Bernstein polynomials.

**Table 2:** Comparison of the AEs for Example 5.2 with $\upsilon (t)=t $

*M*	*t*	Proposed method	Method of [37]
5	0.1	4.39175e − 3	3.17089e − 3
	0.3	1.54003e − 3	4.38223e − 4
	0.5	3.13373e − 4	3.33008e − 3
	0.7	2.45949e − 4	3.03242e − 2
	0.9	2.29803e − 4	2.02549e − 1
6	0.1	2.87601e − 4	1.31233e − 3
	0.3	4.15540e − 5	1.28026e − 4
	0.5	1.33728e − 5	3.72545e − 3
	0.7	8.30744e − 5	2.64696e − 2
	0.9	8.30885e − 5	1.81576e − 1
7	0.1	2.76736e − 6	8.24676e − 4
	0.3	1.16055e − 6	9.11265e − 5
	0.5	1.01844e − 5	1.25615e − 3
	0.7	1.09912e − 5	9.57862e − 3
	0.9	1.10564e − 5	8.65824e − 2

### Example 5.3

Consider the following VO problem [38, 39]:

$$\begin{aligned} {{}^{C}D^{\upsilon (t)}_{t} }z(t)= \frac{2t^{2-\upsilon (t)}}{\Gamma (3-\upsilon (t))}+ \frac{3t^{1-\upsilon (t)}}{\Gamma (2-\upsilon (t))}, \quad t\in [0,1], \end{aligned}$$

where

$$\begin{aligned} z(0)=0. \end{aligned}$$

The analytical solution is $z(t)=t^{2}+3t$. By considering $\upsilon (t)=\sin (t),\frac{t}{2} $ and choosing $M=2$, we get

$$\begin{aligned} z_{0}=\frac{31}{16}\sqrt{\frac{\pi }{2}},\qquad z_{1}=\frac{3\pi }{2}, \qquad z_{2}=\frac{1}{16} \sqrt{\frac{\pi }{2}}, \end{aligned}$$

which gives the analytical solution. As it is seen, the proposed scheme gives the analytical solution with $M=2 $ (only three basis functions) compared to the methods introduced in [38–40]. Table 3 reports the maximum absolute errors (MAE) ($E_{\infty }(M)$) obtained in [38–40].

**Table 3:** Comparison of the MAE $E_{\infty }(M)$ for Example 5.3

*υ*(*t*)	*M*	$E_{\infty }(M)$ ([38])	$E_{\infty }(M)$ ([39])	$E_{\infty }(M)$ ([40])
sin(*t*)	4	2.47e − 2	7.53e − 7	2.87e − 1
	8	5.60e − 3	3.16e − 10	1.44e − 1
	16	1.33e − 3	2.11e − 10	7.26e − 2
$\frac{t}{2}$	4	5.98e − 3	1.20e − 6	2.12e − 1
	8	1.42e − 3	3.34e − 9	1.06e − 1
	16	3.47e − 4	1.08e − 10	5.30e − 2

## Conclusion

In this research, we have generalized a collocation method including the shifted fifth-kind Chebyshev polynomials to numerically solve variable order integro-differential equations in the Caputo sense. For finding approximate solutions of the considered equations, we have used the properties of the shifted fifth-kind Chebyshev polynomials. In addition, by applying the collocation points, we have changed the primary problem to solving a system of algebraic equations to get an approximate solution. Also, we have discussed the convergence of the numerical solution obtained by the proposed scheme. Eventually, the efficiency and suitability of the proposed scheme are displayed by solving some problems of variable order.

## Data Availability

Not applicable.

## References

[1] Podlubny I. (1970). Fractional Differential Equations.

[2] Karapinar E., Binh H.D., Luc N.H., Can N.H. (2021). On continuity of the fractional derivative of the time-fractional semilinear pseudo-parabolic systems. Adv. Differ. Equ..

[3] Ganji R.M., Jafari H., Kgarose M., Mohammadi A. (2021). Numerical solutions of time-fractional Klein–Gordon equations by clique polynomials. Alex. Eng. J..

[4] Adiguzel R.S., Aksoy U., Karapinar E., Erhan I.M. (2020). On the solution of a boundary value problem associated with a fractional differential equation. Math. Methods Appl. Sci..

[5] Afshari H., Karapinar E. (2020). A discussion on the existence of positive solutions of the boundary value problems via *ψ*-Hilfer fractional derivative on b-metric spaces. Adv. Differ. Equ..

[6] Jafari H., Ganji R.M., Nkomo N.S., Lv Y.P. (2021). A numerical study of fractional order population dynamics model. Results Phys..

[7] Afshari H., Kalantari S., Karapinar E. (2015). Solution of fractional differential equations via coupled fixed point. Electron. J. Differ. Equ..

[8] Alqahtani B., Aydi H., Karapinar E., Rakocevic V. (2019). A solution for Volterra fractional integral equations by hybrid contractions. Mathematics.

[9] Karapinar E., Fulga A., Rashid M., Shahid L., Aydi H. (2019). Large contractions on quasi-metric spaces with an application to nonlinear fractional differential equations. Mathematics.

[10] Adiguzel R.S., Aksoy U., Karapinar E., Erhan I.M. (2021). Uniqueness of solution for higher-order nonlinear fractional differential equations with multi-point and integral boundary conditions. Rev. R. Acad. Cienc. Exactas Fís. Nat., Ser. A Mat..

[11] Adiguzel R.S., Aksoy U., Karapinar E., Erhan I.M. (2021). On the solutions of fractional differential equations via Geraghty type hybrid contractions. Comput. Math. Appl..

[12] Ardjouni A. (2021). Asymptotic stability in Caputo-Hadamard fractional dynamic equations. Results Nonlinear Anal..

[13] Ray S.S. (2016). The formation of dynamic variable order fractional differential equation. Int. J. Mod. Phys. C.

[14] Khan M.A., Atangana A. (2020). Modeling the dynamics of novel coronavirus (2019-nCoV) with fractional derivative. Alex. Eng. J..

[15] Ganji R.M., Jafari H., Nkomo N.S., Moshokoa S.P. (2021). A mathematical model and numerical solution for brain tumor derived using fractional operator. Results Phys..

[16] Samko S.G., Ross B. (1993). Integration and differentiation to a variable fractional order. Integral Transforms Spec. Funct..

[17] Lorenzo C.F., Hartley T.T. (2002). Variable order and distributed order fractional operators. Nonlinear Dyn..

[18] Doha E.H., Abdelkawy M.A., Amin A.Z.M., Baleanu D. (2018). Spectral technique for solving variable-order fractional Volterra integro-differential equations. Numer. Methods Partial Differ. Equ..

[19] Luc N.H., Baleanu D., Long L.D., Can N.H. (2020). Reconstructing the right-hand side of a fractional subdiffusion equation from the final data. J. Inequal. Appl..

[20] Soon C.M., Coimbra C.F.M., Kobayashi M.H. (2005). The variable viscoelasticity oscillator. Ann. Phys..

[21] Yang X.J. (2017). Fractional derivatives of constant and variable orders applied to anomalous relaxation models in heat-transfer problems. Therm. Sci..

[22] Neto J.P., Coelho R.M., Valerio D., Vinga S., Sierociuk D., Malesza W., Macias M., Dzielinski A. (2018). Simplifying biochemical tumorous bone remodeling models through variable order derivatives. Comput. Math. Appl..

[23] Moghaddam B.P., Tenreiro Machado J.A. (2017). Time analysis of forced variable-order fractional van der Pol oscillator. Eur. Phys. J. Spec. Top..

[24] Ingman D., Suzdalnitsky J. (2018). Control of damping oscillations by fractional differential operator with time-dependent order. Comput. Methods Appl. Mech. Eng..

[25] Ramirez L.E.S., Coimbra C.F.M. (2007). A variable order constitutive relation for viscoelasticity. Ann. Phys..

[26] Hamoud A., Mohammed N.M., Ghadle K. (2020). Existence and uniqueness results for Volterra–Fredholm integro-differential equations. Adv. Theory Nonlinear Anal. Appl..

[27] Ganji R.M., Jafari H., Adem A.R. (2019). A numerical scheme to solve variable order diffusion-wave equations. Therm. Sci..

[28] Ganji R.M., Jafari H., Baleanu D. (2020). A new approach for solving multi variable orders differential equations with Mittag-Leffler kernel. Chaos Solitons Fractals.

[29] Ray S.S. (2021). A novel wavelets operational matrix method for the time variable-order fractional mobile-immobile advection-dispersion model. Eng. Comput..

[30] Ray S.S. (2021). A new approach by two-dimensional wavelets operational matrix method for solving variable-order fractional partial integro-differential equations. Numer. Methods Partial Differ. Equ..

[31] Tuan N.H., Ganji R.M., Jafari H. (2020). A numerical study of fractional rheological models and fractional Newell–Whitehead–Segel equation with non-local and non-singular kernel. Chin. J. Phys..

[32] Jafari H., Tuan N.H., Ganji R.M. (2021). A new numerical scheme for solving pantograph type nonlinear fractional integro-differential equations. J. King Saud Univ., Sci..

[33] Jafari H., Ganji R.M., Sayevand K., Baleanu D. (2021). A numerical approach for solving fractional optimal control problems with Mittag-Leffler kernel. J. Vib. Control.

[34] Almeida R., Tavares D., Torres D.F.M. (2019). The Variable-Order Fractional Calculus of Variations.

[35] Abd-Elhameed W.M., Youssri Y.H. (2017). Fifth-kind orthonormal Chebyshev polynomial solutions for fractional differential equations. Comput. Appl. Math..

[36] Jafari H., Tajadodi H., Ganji R.M. (2019). A numerical approach for solving variable order differential equations based on Bernstein polynomials. Comput. Math. Methods.

[37] Yi M., Huang J., Wang L. (2013). Operational matrix method for solving variable order fractional integro-differential equations. Comput. Model. Eng. Sci..

[38] Cao J.X., Qiu Y.N. (2016). A high order numerical scheme for variable order fractional ordinary differential equation. Appl. Math. Lett..

[39] Li X., Li H., Wu B. (2017). A new numerical method for variable order fractional functional differential equations. Appl. Math. Lett..

[40] Shen S., Liu F., Chen J., Turner I., Anh V. (2012). Numerical techniques for the variable order time fractional diffusion equation. Appl. Math. Comput..

